# Considerations for oral and dental tissues in holistic care during long-haul space flights

**DOI:** 10.3389/fphys.2024.1406631

**Published:** 2024-07-11

**Authors:** Mahmoud M. Bakr, Gabrielle M. Caswell, Habiba Hussein, Mohamed Shamel, Mahmoud M. Al-Ankily

**Affiliations:** ^1^ School of Medicine and Dentistry, Griffith University, Gold Coast, QLD, Australia; ^2^ Spaceport Australia, Moree, NSW, Australia; ^3^ Faculty of Dentistry, The British University in Egypt, Cairo, Egypt

**Keywords:** bone density, oral microbiology, dental care, microgravity, space travel, *Streptococcus mutants*, xerostomia

## Abstract

The health of astronauts during and after the return from long-haul space missions is paramount. There is plethora of research in the literature about the medical side of astronauts’ health, however, the dental and oral health of the space crew seem to be overlooked with limited information in the literature about the effects of the space environment and microgravity on the oral and dental tissues. In this article, we shed some light on the latest available research related to space dentistry and provide some hypotheses that could guide the directions of future research and help maintain the oral health of space crews. We also promote for the importance of regenerative medicine and dentistry as well highlight the opportunities available in the expanding field of bioprinting/biomanufacturing through utilizing the effects of microgravity on stem cells culture techniques. Finally, we provide recommendations for adopting a multidisciplinary approach for oral healthcare during long-haul space flights.

## Introduction

Space flights have a significant effect on different tissues of the body (Iwase et al., 2020) and the oral cavity (Mednieks and Hand, 2019) including the nervous system (Onorato et al., 2020), musculoskeletal system (Juhl IV et al., 2021); bone (Stavnichuk et al., 2020), teeth (Moussa et al., 2023), saliva (Sun et al., 2022) and skin (Caswell and Eshelby, 2022). The majority of the changes are related to the changes in microgravity, increased exposure to radiation in particular the galactic cosmic rays (GCR), using dry washing technique, variations in air quality and changes in microbe replication and growth parameters which could be related to immunosuppression (Onorato et al., 2020; Stavnichuk et al., 2020; Juhl IV et al., 2021; Caswell and Eshelby, 2022; Sun et al., 2022). Furthermore, the interaction between microgravity and the exposure to ionizing radiation could have a substantial effect on physiological events and cellular responses (Yatagai et al., 2019).

Dental emergencies have a substantial impact on space missions to the extent that it has been classified as one of the top five essential conditions that need to be predicted before launching a space mission (Menon et al., 2012). As a result, The Space Medicine Exploration Medical Condition List included a number of dental conditions not limited to toothache, pulpitis, avulsion, tooth loss, crown replacement, and temporary fillings (Watkins et al., 2011; Johnston et al., 2014; Blue et al., 2019). More recent, a new medical conditions list was developed for space exploration flights based on reviewing information from nine previous lists in a systematic way by subject matter experts based on their history of occurrence in previous flights and citation in previous lists (Kreykes et al., 2023). It would not be a surprise to mention that dental conditions were a priority on the list. The above work related to medical conditions list relevant to space flights would help in reduction of the number of dental events. However, it should be taken into consideration that other physiological changes in the body due to the influence of microgravity (bone loss as an example), could indirectly result in dental disease and/or a relevant dental event during space flights as a result of upsetting the balance within other oral anatomical structures, saliva or oral microbiome (Lloro et al., 2020).

## The effect of space flights on dental caries

### Factors affecting dental caries progression in space

There are several factors that contribute to the development of relevant dental events in space. These factors include, increase in salivary immunogolbulins (Brown et al., 1974; Brown et al., 1976) and increase of salivary Mycoplasms (Brown et al., 1974). In longer space flights, anaerobic bacteria populations increased even in studies with simulated microgravity (Brown et al., 1976). Changes in the oral microbiome could be related to the lack of a balanced diet during space exploration missions (Tang et al., 2021) as well as the stress that astronauts have to endure. This highlights the importance of establishing sustainable food production systems (Tang et al., 2021; Pandith et al., 2023) and the mental wellbeing with regards to psychological support to astronauts (Arone et al., 2021; Oluwafemi et al., 2021). Furthermore, there is growing evidence that cosmic radiation during space travel alters gut microbiota including the *Lactobacillus* and *Bacteroides* families (Li et al., 2022). We hypothesize that the oral microbiome is also affected by cosmic radiation which could lead to accelerating the progression of dental caries and other forms of oral diseases through a number of mechanisms including the alteration of the immune response and mutation accumulation. The changes in the oral microbiome should not be taken lightly as it would lead to a state of chronic inflammation that could be a risk factor for increasing the chances of the incidence of other chronic conditions such as asthma, arthritis and ischemic heart disease (Lloro et al., 2020). Furthermore, the state of chronic inflammation in the oral cavity, increases the permeability of mucous membranes that could act as potential entry routes for microbes through the oral cavity, eyes, nose and respiratory tracts (Szot et al., 2017; Gomes et al., 2018).

### Possible mechanisms of dental caries development and progression during space flights

There have been cases of dental pain related to caries progression during space flights reported with no success of identifying the origin of the problem (Gontcharov et al., 2005; Menon, 2012). It is understandable that progression of the caries would be related to the increase in anaerobic bacteria as described above and the elevated *Streptococcus mutants* count (Orsini et al., 2017) as well as the variations/evolution of *S. mutants* species that alters their survival and pathogenic properties (Fernander et al., 2022; Fernander et al., 2023). However, the origin of the carious lesion could have been an area of pre-existing demineralization or arrested caries. This raises the point of carefully selecting astronauts during the recruitment process and ensuring that all pre-existing lesions are treated as well as preventative measures applied to avoid caries progression. It could be argued that the increase in IgA and IgG discussed above could have contributed to increasing the adhesion of cariogenic substances and microbes to the salivary biofilm on the teeth surfaces (Costalonga and Herzberg, 2014). In addition to the above, studies have shown increased levels of glucosyltransferase enzymes during microgravity adding strength to the virulence of *S. mutants* through facilitating the synthesis of glucans from sucrose and mediating the attachment of micro-organisms to tooth surfaces (Omar et al., 2012).

### Effects of microgravity on dental caries progression

Further investigating the hypothesis of increased virulence, gene expression and antibiotic resistance of pathogens in simulated microgravity (Taylor, 2015; Senatore et al., 2020) is not only important for astronauts but also is significant for understanding and exposing potential alternative mechanisms of disease progression that could be applied to clinical practice on earth (Salavatifar et al., 2023). In simulated microgravity, increased pain scores around the lower jaw especially around the submandibular and sublingual areas during mandibular movements could be related to the lack of resistance from gravity and the potential loss of muscle tone/strength during long term space flights which could eventually lead to changes in occlusion and/or teeth clashing in occlusal positions that are less ideal secondary to the muscular changes. Furthermore, the sleep disturbances and unregulated sleep patterns of astronauts could also contribute to the oral and dental issues described above as sleep deprivation is linked to higher levels of blood cortisol and elevated levels of stress which would result in physiological changes to the symbiosis of the oral cavity (Pavy Le-Traon and Roussel, 1993; Wu et al., 2018).

## The effect of space flights on dental hard tissues

### Effect of microgavity on craniofacial structures

Understanding the effects of microgravity on craniofacial structures is crucial to predict the risks during long-haul space flights in order to develop comprehensive plans for prevention and management of dental emergencies (Moussa et al., 2023). However, it has been noted that there is not a sufficient number of studies that discuss the effects of the space environment on many areas of the craniofacial complex such as the maxilla, temporomandibular joint, molar, premolar, and canine teeth, as well as small sample sizes for the studies of the mandible and incisors (Moussa et al., 2023). There are indications that space flights might induce changes in the teeth/dentition, for example, it was shown that the pulpal size increased in space flight animals which indicates deficiency in dentin formation and/or quality (Maupin et al., 2019). Furthermore, there were contradictory results related to incisor calcium (Ca) and phosphorus (P) content between studies conducted during the Cosmos-1887 mission (Simmons et al., 1990) and the Cosmos-1129 mission (Rosenberg et al., 1984). In the first study, the Ca and P content remained relatively unchanged (Simmons et al., 1990), while in the second study, the overall Ca and P content in the entire dentin was greater in space flights compared to ground crew samples but in the inner half of dentin (formed during the space flight) was relatively deficient (Rosenberg et al., 1984). It should be noted that according to our knowledge, no studies were conducted so far on the effect of cosmic radiation on dental hard tissue. We hypothesize that the exposure to higher radiation doses during long-haul space flights will negatively impact dental hard tissues based on the previous knowledge related to the development of radiation caries after the exposure to high doses of radiation during radiotherapy for cancer treatment which could change the mechanical properties, ultrastructure, appearance, crystal properties, and the chemical composition of dental hard tissue (Lu et al., 2019).

### Directions for future research on the effect of space flights on teeth

With the limited data available on the effect of space flights on teeth and dental hard tissues, the results remain unconclusive. This is due to the absence of consistency between different studies where different factors were not standardized nor considered such as the duration of the space flight, diet, age and sex of the samples in each study (Moussa et al., 2023). Furthermore, most of the studies conducted on the effect of space flights on teeth/dental hard tissues used rodents which are completely different from humans that their incisors continuously erupt, the tooth enamel and dentin are constantly deposited by ameloblasts and odontoblasts (Goldberg et al., 2014). This makes the translation of results into human subjects inaccurate and unreliable. However, rodents’ molar teeth show limited continuous eruption and renewal of their dental tissues which can be a more accurate comparison to human teeth in response to environmental factors and space conditions including microgravity (Goldberg et al., 2014; Moussa et al., 2023). More studies are necessary to develop valid conclusions with regards to the effects of long-haul space flights on dental hard tissue and teeth in order to fill the current gaps in the literature and knowledge.

## The effect of space flights on the alveolar bone

### Effect of microgravity on bone loss

Cosmonauts experience three stages of physiologic adaptation resulting from altering gravity during space flights: 1) alterations upon entering weightless (initial adaptation), 2) modifications during extended time in weightlessness, and 3) re-adaptation to Earth’s gravity (Iwase et al., 2020). The immuno-haematological, bone metabolic, musculoskeletal, cardiovascular and neurovestibular systems of the body are each affected by weightlessness; alterations to these systems occur during these adaptation periods (Frippiat et al., 2016; Iwase et al., 2020; Tesei et al., 2022).

Loss of bone due to exposure to microgravity is a serious problem for the wellbeing of crew members on exploration flights (Oganov et al., 1991; Leblanc et al., 2000; Vico et al., 2000; Smith and Heer, 2002; Smith et al., 2005). Exposure to weightless causes bones’ fragility, which can affect them even after their return (Planel, 2004; Iwase et al., 2020). Although the exact process of loss of bone mineral during space missions is unknown, obviously it is multi-factorial (Zwart et al., 2018). Microgravity has significant negative consequences such as muscular atrophy and, more crucially, loss of bone. Throughout a mission to space, individuals lose a mean of between 0.5 and 2 percent of bone mass each month, or six to twenty-four percent annually (Hodkinson et al., 2017; Lloro et al., 2020; Stavnichuk et al., 2020; Moroni et al., 2022). Plenty of research indicate that weightless can induce osteoclastogenesis while increasing resorption of bones, either during long-haul space flights or in a simulated microgravity setting (Lloro et al., 2020).

### Pharmacological approaches for a favourable bone response during space flights

As the density of bones is adequate before the commencement of flight, the best approach to pharmacotherapy preventing loss of bone is to avoid loss of bone rather than accelerating its production once stress is reduced throughout flight. Multiple medications have been suggested to assist with avoiding loss of bone in weightless (Iwase et al., 2020). Bisphosphonates possess two phosphonate groups as well as are structurally identical to pyrophosphate. They attach to hydroxyapatite in the bone matrix, preventing loss of bone by blocking osteoclastic resorption of bone. In addition, these medications have been shown to be beneficial for inhibiting the loss of bones throughout bedrest trials (Thompson et al., 1990; Grigoriev et al., 1992; Rodan and Fleisch, 1996; Iwase et al., 2020). Despite the inherent risk of bisphosphonate related osteonecrosis of the jaw (BRONJ) that is associated with bisphosphonate treatment, the risk is minimal during space travel due to the strict requirements and screening that astronauts go through before being accepted in the space crew. This minimizes the chances of needing invasive procedures that involve manipulation of the jawbone during space missions.

Amongst numerous forms of bisphosphonates, pamidronate has been proved to slow bones’ minerals loss and avoid the production of renal stones throughout bed rest trials (Watanabe et al., 2004; Iwase et al., 2020). In summary, the majority of the bone degradation seen in the astronauts who took part in this investigation may have non-reversible characteristics. Even though bisphosphonates, medications that prevent resorption of bone and are employed in conjunction with physical activity, have been demonstrated to be effective in maintaining DXA-BMD at the spine and hip levels, the reported variability in bone response pattern could result in unexpected treatment outcomes (LeBlanc et al., 2013). Although bisphosphonates and nutrition were regarded as therapeutic options to reduce loss of bone, exercising proved as the most effective (LeBlanc et al., 2002; LeBlanc et al., 2013; Hodkinson et al., 2017). As such, resistance exercise, particularly, is considered to promote osteogenesis (Guadalupe-Grau et al., 2009; Hodkinson et al., 2017).

### Effects of microgravity and space travel on the mandible

Previous research investigated the implications of space travel and simulated microgravity on the mandible. In rats flown aboard Cosmos 1129 for 18.5 days, periosteal osteogenesis decreased in non-muscle-covered parts of the mandibular bone (molar area) (Simmons et al., 1990). The mesial portion of the first molar had less alveolar bone formation, suggesting a delay of mouse molars’ usual distal drift. There was also a reduction in alveolar bone mineral and collagen in the most matured parts, but elevated levels in the most undeveloped parts, indicating a delay in maturity (Simmons et al., 1990). The lower jaws of rats that travelled for a 12-day period aboard Cosmos 1887 exhibited comparatively elevated calcium and magnesium levels, even though their hydroxyapatite crystals were smaller (Simmons et al., 1990; Mednieks and Hand, 2019). Changes in bone architecture and blood vessels are less pronounced in the lower jaw than in the vertebra and the lower extremities’ long bone. In the Bion-M1 flying and habitat control mice, the reduction in blood vessels observed was probably attributable their consumption of soft paste food. Flight mice have a greater percentage of sclerostin-positive osteoclasts and osteocytes in their lower jaws, indicating that weightlessness might have a role (Mednieks and Hand, 2019).

Other animal and human investigations that extended multiple weeks indicated that reducing mastication and hence occlusal pressures influenced all tissues, such as bones and musculature (Philippou et al., 2015; Treffel et al., 2016). A different possible worry is osteonecrosis of the maxilla and mandible, but the likelihood is limited (Woo et al., 2005; Gill et al., 2009; Iwase et al., 2020). It should be noted that the issue of possible osteonecrosis is not related to space flights or microgravity but rather as a result of the side effects of the use of bisphosphonates. These osteolytic or osteonecrotic occurrences consistently coincide with tooth infection, iatrogenic trauma (tooth extraction/denture damage), or physiological strain (mastication) (Ruggiero et al., 2004; Ruggiero and Woo, 2008; Iwase et al., 2020). It is difficult to hypothesize based on the available animal studies (Ghosh et al., 2016; Moussa et al., 2023) that the alveolar is bone would be significantly impacted at a similar capacity in long-haul space flights as other bones in the body. Therefore, further research is deemed necessary to confirm the extent of the well-established negative effects of microgravity on skeletal bones and its correlation with the maxillary and mandibular alveolar bone.

## The effect of space flights on the oral mucosa

### Effect of salivary changes on the oral mucosa during space flights

Oral mucosa is the protective intra-oral layer that acts as barrier against oral infection as well as contributing to wound healing. It is always laminated by a thin film of saliva that keeps it wet. It has been demonstrated in literature that the salivary composition changes under simulated microgravity conditions (Sun et al., 2022). Furthermore, changes in the size of salivary glands, reduction in masticatory activity and changes in the expression of salivary proteins are also evident (Mednieks and Hand, 2019). To be more specific, it has been noted that simulated microgravity results in the shift in the salivary microbiome from oral health related bacteria to oral disease related bacteria with a trend towards reduction of the salivary PH which still remained alkaline (Sun et al., 2022). Another study revealed that salivary microbiome changes during space flights are correlated with viral reactivation of certain conditions that affect the oral mucosa (Urbaniak et al., 2020).

### Oral mucosal conditions during space travel and their effect on the overall health

Multiple oral mucosa related issues have been associated with long-haul space flights and prolonged exposure to microgravity including sarcopenia (Runfeldt et al., 2019), peripheral nerves alterations, neuromotor plaque in the muscles of mastication, labial, lingual and buccal weakness, nociplastic pain in oral mucosal diseases, soft tissue changes and pathologies related to chewing, corticomotor neuroplasticity of tongue, and swallowing biomechanics (Dugan et al., 2023). Various modern techniques including transcranial electrical and magnetic stimulation (tES and TMS) which are non-invasive brain simulation techniques that are used in pain control and could be helpful in space flights (Ekhtiari et al., 2019). Furthermore, it has been proven that the space flights conditions had an impact on the overall microbiome of astronauts and the host-microbe interactions (Tesei et al., 2022). Probiotics have been suggested as promising countermeasures for health issues during long-haul space flights and we propose that this could be applied for oral diseases and infections (Bharindwal et al., 2023). Further studies are deemed necessary to validate this proposal.

### Preventative measures against soft tissue infections during space flights

Due to the potential of an immune system drop (Sonnenfeld, 2005; Crucian et al., 2008) and delayed wound healing (Davidson et al., 1998) during long-haul space flights, every effort should be made to minimize the chances of infection in human space travel. Exercise (Shearer et al., 2009), vitamin D supplements (Martineau et al., 2011), maintaining a healthy microbiological status (Ilyin, 2005), waterless hand hygiene products, personal protective equipment, chlorhexidine mouth rinses, gamma irradiation of selected foods and ultraviolet light for disinfecting surfaces (Mermel, 2013) are highly important due to the increased chances of opportunistic infections in space. An interesting finding from a recent study showed that *Bacillus horneckiae*, that was first identified within the Kennedy Space Centre was present in the impression disinfection solution in a university clinic (Chukwu et al., 2024). This bacterial species was not killed with the industry standard impression disinfectant solution and its pathogenicity is still unknown (Chukwu et al., 2024). This highlights the high variability in bacterial species during long-haul space flights and the need for strict infection control/prevention measures during space flights.

## The effect of space flights on periodontal disease

### Microgravity as a risk factor for periodontal disease

The revelation that periodontal issues are the next most prevalent dental disease in prolonged solitary settings implies that such long duration has an impact on oral hygiene, however, it is reported that the increased risk for periodontal disease in long-haul space flights is mainly related to less than optimal oral care, environmental factors as the dry and cold air in spaceships as well as the lack of pre-mission visits to the dentist for regular check-ups and regular professional dental care (Kupper et al., 2014). That scenario might be readily resolved by teaching the aboard medical staff on dental hygiene using an ultrasonic scaler and demanding everyone onboard to get regular professional hygiene (Lloro et al., 2019). In addition, low salivary flow and dry mouth increase the risk of caries and periodontal disorders; dry mouth can be caused by inhaling dry compressed gases in an aeroplane (Fontana and Zero, 2006; Pathak, 2015).

Microgravity and low-shear stress can cause an imbalance in microbial communities and alter bacterial physiological processes (Nickerson et al., 2000; Nickerson et al., 2004; Wilson et al., 2007; Castro-Wallace et al., 2017; Fernander et al., 2022). Oral occurrences reported from lengthy as well as weightless operations included a rise in streptococcal and anaerobic components (*Streptococcus mutans*) in saliva and dental plaque microorganisms in addition to a growth in IgA in saliva as well as demonstrating an elevated prevalence over brief durations (Lloro et al., 2020). The gastrointestinal tract is the most complex community followed by the human mouth which has over 1000 distinct species (Dewhirst et al., 2010; Human Microbiome Project Consortium, 2012; Fernander et al., 2022). Studies have demonstrated that microorganisms can create biofilms, produce extracellular polysaccharides, grow faster, change their pathogenic stress response and virulence, acquire new resistance, and produce more secondary metabolites when exposed to simulated microgravity (Fang et al., 1997; Nickerson et al., 2000; McLean et al., 2001; Brown et al., 2002; Lynch et al., 2004; Allen et al., 2008; Crabbé et al., 2008; Mauclaire and Egli, 2010; Castro et al., 2011; Gilbert et al., 2020; Fernander et al., 2022).

### Biofilm considerations for periodontal disease during long-haul space flights

Despite environmental stresses such as eating habits, pH, temperature, and flow of saliva, bacterial communities may maintain the density of cells of up to 100 CFU mL (Evaldson et al., 1982; Kolenbrander et al., 2006; Palmer et al., 2006; Kreth et al., 2008; Fernander et al., 2022). Oral microorganisms contribute to both systemic and oral health because bacteria live on all of the oral cavity’s surfaces (teeth, tongue, gums, etc.), blocking pathogen colonization. (Shroff et al., 1995; Hooper et al., 2012; Fernander et al., 2022). Nevertheless, tooth caries is still amid the most common disorders among people (Takahashi and Nyvad, 2011; Fernander et al., 2022). The phenotypes of the bacteria causing periodontal disease on earth are extensively known, and treatment options are more predictable, but this may not be the case during long-term space flight (Marsh, 1999; Schachtele et al., 2007; Gross et al., 2010; Fernander et al., 2022). A simulated study of a Skylab mission found that secretory immunoglobulin A levels steadily rose in chamber confinement, peaking at the 55th day of monitoring. Though the alterations in enteric bacilli and *mycoplasma* were statistically noteworthy, there was not a spike in *Streptococcus mutans* in saliva. Nevertheless, a study showed a rise in *Streptococcus mutans* in plaque on teeth, which was linked to a diet high in sugar (Brown et al., 1974; Lloro et al., 2020).

*Bacteroides species*, *Veillonella species*, *Fusobacterium species*, *Neisseria species*, and *Streptococcus mutans* appear in larger quantities in periodontitis when compared to the healthy status of the oral cavity (Costalonga and Herzberg, 2014). These bacterial species were present and proliferated in the oral microbiome of the astronauts over three Skylab missions (Lloro et al., 2020). *Veillonella species* in plaque might account for as much as 45 percent throughout the early phases of periodontal or gingival inflammation. In addition to other risk factors, *Fusobacterium species* is a direct bacterial pathogen that contributes to the development of several periodontal disorders (Ardila et al., 2010; Lloro et al., 2020)*. L. salivarius*, *P. denticolens*, *S. mutans* and *S. wiggsiae* were nearly entirely detected in plaques derived from dentinal caries. However, in infants, S. sanguinis and some species of *Neisseria* and *Leptotrichia* were regularly discovered in healthy tooth’s plaque (Richards et al., 2017). Similar bacterial strains were evident under the effect of microgravity, but the question remains on whether the changes in the oral microbiome is solely related to the effect of microgravity or other factors as radiation, isolation, stress which also affect oral and systemic health in space (Lloro et al., 2020). The significance of the development of periodontitis after bacterial proliferation is clear given that, in an individual suffering from a mild periodontal inflammation, the portion of the periodontal bag directly contacting the bacterial plaque is around 72 cm^2^, which is roughly the human palm’s size (Page, 1998; Lloro et al., 2020). Table 1 summarizes the bacterial species found during space missions and their role in development of dental diseases on earth.

**TABLE 1:** A summary of the bacterial phenotypes isolated from different space missions and their origin as well as role in dental and/or periodontal disease on earth.

Bacterial species	Effect on the teeth and periodontium
*P. denticolens*	Common in dental caries
*Fusobacterium species*	Contributes to the development of several periodontal disorders
*Leptotrichia species*	Found in plaque around healthy teeth in infants
*S. mutans*	Common in dental caries
*Neisseria species*	Found in plaque around healthy teeth in infants
*L. salivarius*	Common in dental caries
S. *sanguinis*	Found in plaque around healthy teeth in infants
*Veillonella species*	Contributes to early phases of gingival and periodontal inflammation
*S. wiggsiae*	Common in dental caries

### Mechanism of development and progression of periodontal disease in space travellers

A comprehensive evaluation of the influence of periodontal condition on cortisol levels in saliva demonstrated its rise proportional with the advancement and extent of periodontitis (Botelho et al., 2018; Lloro et al., 2020). Cortisol is an oxidative damage mediator; hence it could be contributory to these variations as oxidative stress appears to play a significant role in advanced periodontal inflammation (Aschbacher et al., 2013; Acquier et al., 2017; Lloro et al., 2020). Based on these findings, a study determined that adding cortisol to the culture media at 12 and 24 hours drastically boosted the development rate of *Porphyromonas gingivalis* dose-independently. This study indicates that cortisol could have a particular influence on the development of *Porphyromonas gingivalis* (Akcali et al., 2014; Lloro et al., 2020). This aligns with recent literature indicating that elevated cortisol levels have a significant effect on the immune system’s response in particular T-cells (Miranda et al., 2023) and antimicrobial salivary proteins (Agha et al., 2020). However, it should be noted that other studies indicated that different space flight stressors (rather than cortisol alone) may interact and produce adverse health effects amongst space travellers (Radstake et al., 2022). This area of research needs further exploration as cortisol levels fluctuate during space flights as a result of altered sleep patterns (Gemignani et al., 2014). We propose that further space environment simulation studies are necessary to understand the full effect of different stressors (including cortisol) on the overall physical and mental health of astronauts. We recommend that collecting salivary samples to measure cortisol levels would be a convenient tool for sample collection.

Considering how alterations in cosmonaut physiological processes impact periodontal state, it is critical to emphasize its biologic link with systemic illnesses such as obstetric difficulties, cardiovascular disease, diabetes, chronic respiratory diseases, and cancer (Akcali et al., 2014; Bui et al., 2019; Lloro et al., 2020). The research extensively reports a link amongst periodontitis, aging, and chronic non-communicable illnesses (Bui et al., 2019). A consistent theme that arose was the effects on low-grade systemic inflammation caused by periodontal bacteraemia/endotoxemia caused by daily activities such as eating and teeth brushing via acute-phase (C-reactive protein, CRP) and neutrophil oxidative stress responses (Sanz et al., 2018; Lloro et al., 2020).

Preventive measures and prior investigations significantly reduce dental incidents. This underscores the need for specialized dental staff and supplies during unique situations, particularly in upcoming long-term space trips (Lloro et al., 2019). To minimize the chance of contracting infections in space, both the team and the spacecraft are subjected to extensive microbial screening before the mission. However, this precautionary measure might not avoid new evolving characteristics from emerging within the member’s microflora because of being subjected to the new conditions found during long-haul space flights (Barratt and Pool, 2008; Fernander et al., 2022).

There are limited studies that discuss the effect of space flights on periodontal disease. However, several studies have shown that motility and chemotaxis of some bacterial strains increased under microgravity conditions (Acres et al., 2021). Furthermore, it was reported that other environmental and genetic host-related factors increased the chances of biofilm formation (Rossi et al., 2018), affected oxygen availability (Marteyn et al., 2011), increased adhesion and invasion of epithelial cells (Lee and Falkow, 1990). The above changes were attributed to the downtrend of the regulation of expression of *hfq* which is an important post-transcriptional factor that facilitates the pairing of small RNAs with their target mRNAs with an important role in bacteria (Valentin-Hansen et al., 2004; Gottesman and Storz, 2011; Vogel and Luisi, 2011). Decreased *hfq* expression was evident in simulated microgravity studies and consistent with stress response studies (Gangaiah et al., 2014; Kim et al., 2015; Wang et al., 2018). We cannot exclude the possibility of ionizing/cosmic radiation affecting the microbiome via mutation accumulation. One of the under-rated biological hazards of ionizing radiation during space travel is microbiome-mediated pathophysiology (Casero et al., 2017), which warrants further investigations to establish the chronic effects of radiation on the oral health. Most of the available literature is related to gut microbiota rather than the oral microbiome and is mainly based on animal models (Fernandes et al., 2023).

In addition to the bacterial-related factors, we believe that the changes in bone and salivary microbiome discussed above as well as the potential drop in immunity as result of prolonged exposure to space radiation and microgravity (Cao, 2022) could contribute to the development of periodontal disease during long-haul space flights. It has been reported that simulated microgravity increased the salivary levels of *Actinomyces* species which has a high affinity to sticking on the tooth and root surfaces as well as high association with periodontal disease and apical periodontitis (Do et al., 2017; Sun et al., 2022). Furthermore, it has been shown that the salivary microbiome changes during space flights with *Streptococcus mutants* being the most abundant species contributing to 8% of the total micro-organisms detected (Urbaniak et al., 2020). This supports our hypothesis that the incidence of periodontal disease would tend to increase during long-haul space flights. Furthermore, the reason behind the scarcity of studies around periodontal health in space could be related to the fact that all astronauts undergo a rigorous dental check-up before embarking on space flights to ensure that their dental and periodontal tissues are in a meticulous condition to avoid the need for emergency treatment during long-haul space flights. As a result, all astronauts are selected with no existing ongoing chronic dental and/or periodontal conditions.

## Regenerative medicine and dentistry and space flights

### Challenges and advancements in bioengineering research in space

The lengthy space flights could result in progressive tissue degradation and an increased susceptibility to injury. Therefore, the field of regenerative dentistry, medicine, surgical repair and advancement of wound healing is highly important (Iordachescu et al., 2023). Due to isolation, distance, and the absence of a possible emergency evacuation plan in space, the design of facilities, infrastructure and the specialized equipment required remain a challenging task to the rapidly developing field of regenerative bioengineering (Iordachescu et al., 2023). In addition to the above, the exposure to extensive cosmic radiation complicates the requirements for development of research facilities in during space missions (Röstel et al., 2020).

Despite the obstacles mentioned in the previous paragraph, developments are ongoing in the fields of research of stem cells (Blaber et al., 2014), spheroids - a form of spherical agglomerates of cells (Pietsch et al., 2017), organs-onchips (Low and Giulianotti, 2019) and the implementation of biomanufacturing/bioprinting (Cubo-Mateo et al., 2020; Sharma et al., 2022). Examples of the progress in the field of stem cell research on earth are some FDA-approved therapeutic products including cultured epidermal autografts (Epicell^®^) that allows treating deep dermal burns (Brockmann et al., 2018), or the autologous cultured chondrocytes on porcine collagen membranes (MACI^®^) for fixing cartilage defects (Bliley et al., 2022). There are limitless applications for biofabrication techniques in space so that space flights become self-sufficient (Moroni et al., 2022).

### Tissue engineering and space travel

While microgravity could be viewed as a hurdle for tissue engineering, however, it is a valuable tool that can be utilized in cell culture techniques especially the stem cell culture environment for cell-based therapy (Imura et al., 2019). Furthermore, bone marrow stromal (BMSCs) and bone-derived mesenchymal stem cells that were cultured under simulated microgravity environments had great success in treating different spinal cord and brain injuries through its neuroprotective properties (Yuge et al., 2011; Mitsuhara et al., 2013; Otsuka et al., 2018). In addition to the above, differentiation and growth of dental pulp stem cells was enhanced in animal model that used simulated microgravity (Li et al., 2017) and the 3D growth and architecture of ameloblast-like cells as well as periodontal ligament cells engineering were improved under the microgravity conditions (Pandya et al., 2021). This could open the horizon for rapid developments in the field of regenerative dentistry and tooth vitality. Finally, in order to fully understand best possible ways to overcome the effect of microgravity and galactic cosmic rays (GCRs) on different tissues including the nervous system, the development of animal experimental models could be an alternative method to overcome the challenges described above and accelerate the development of knowledge in the field of regenerative medicine/dentistry and bioengineering in space missions (Onorato et al., 2020). The outcomes from human research conducted in space are of extreme scientific/biomedical importance and translate to medical care on Earth (Shelhamer et al., 2020).

## A multidisciplinary approach to maintaining oral health during space flights

### The importance of treatment planning in aerospace dentistry

Dentists are increasingly concerned about dental issues in solitary and confined groups, particularly those who are isolated or have no access to healthcare. This is especially important given the growth of manned space programs, Antarctica research, and submarine operations. Financial expenses for treatment, specialized dental staff, evacuation, and transportation to dental institutions vary based on the frequency of acute dental occurrences and the dental care provided (Lloro et al., 2019). Therefore, dental planning is crucial for maintaining aircrews’ operational health, especially at higher altitudes, and ensuring oral health to enable uninterrupted tasks (Pathak, 2015; Pathak et al., 2015; Lloro et al., 2019). Aerospace dentistry focuses on enhancing the dental and oral hygiene of crew members, particularly in preventing illnesses caused by changes in atmospheric pressure (Pathak, 2015; Pathak et al., 2015). High-altitude fluctuations can cause discomfort, pain, and organ malfunction like periodontitis, dental abscesses, and deep carious lesions, and for those reasons, a proper assessment could help in preventing them (Pathak, 2015; Pathak et al., 2015).

NASA’s mission to establish human settlement on Mars is a growing concern due to potential risks to mission goals; one of which is that crew members face microbiological issues during long-haul space flights, potentially limiting their production and the ships’ integrity. To minimize infection, extensive microbial screening is conducted (Fernander et al., 2022) The implementation of prevention strategies and prior screenings significantly reduce oral issues rates of occurrence (Lloro et al., 2019). However, the crew’s microflora may develop unique evolutionary traits due to their exposure (Fernander et al., 2022; Fernander et al., 2023). NASA has developed tight requirements for cosmonaut choosing, continuation, as well as prior to flight oral examinations for particular space flights, plus a rigid clinical regimen. Astronauts undergo rigorous screenings 24 weeks before departure, including dental care at least 12 weeks before missions.

### Dental emergencies during long-haul space flights

**FIGURE 1:**
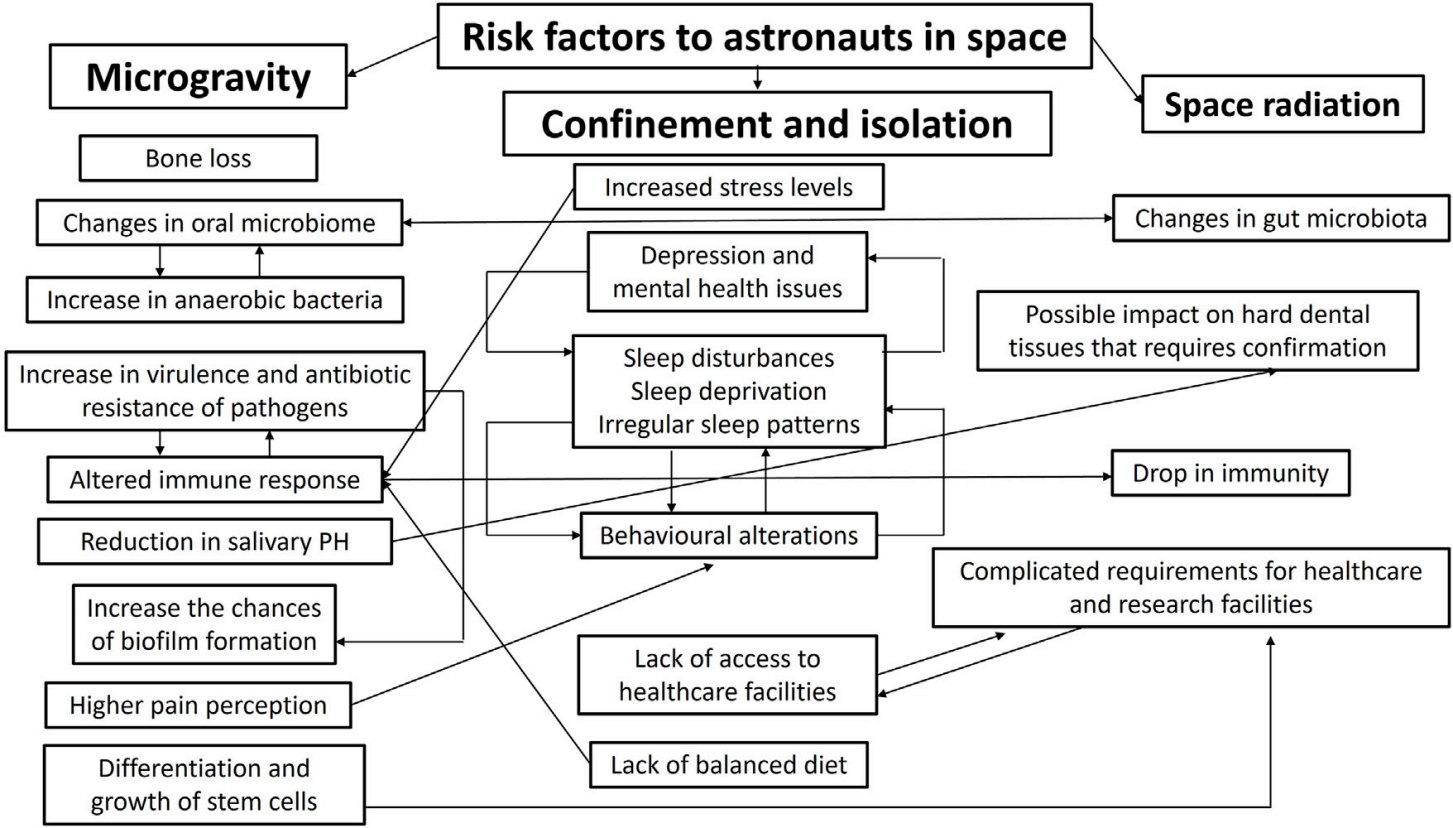
Summarizes the possible correlations between risk factors to astronauts in space and illustrates the complex nature of the health-related challenges in long-haul space flights.

The Space Medicine Exploration Medical Condition List states that basic dental treatments and diagnoses will be accessible (Blue et al., 2019); furthermore, dental emergencies were forecasted to be one of the leading circumstances influencing subsequent mission goals by the Integrated Mathematical Medical Model (Fernander et al., 2022). As time progresses, space medicine is achieving more recognition as a speciality that is involved in different phases of the space flight from the crew selection, training and the space flight itself to post-flight rehabilitation and long-term health of astronauts (Hodkinson et al., 2017). Table 2 summarizes the possible dental emergencies that can occur during as well as before and after space flights. This is extremely important given the complexity of the nature risk factors that astronauts are exposed to during long-haul space flights (Figure 1).

**TABLE 2:** Presenting a summary of the possible dental emergencies that require medical attention during as well as before and after space flights.

In-flight dental emergencies	Near flight (pre- and post-flight) dental emergencies
Crown displacement	Pulpitis
Lost fillings	Displaced crown
Dental pain	Tooth fracture
Dental caries	Periapical abscess
Barodontalgia	Deteriorating amalgam restoration
Avulsion/tooth loss	Loose tooth requiring stabilisation

A dentist would be crucial during long-term space trips to Mars, as full dental treatment, radiographs, and root canals are unavailable in space. Aeronautic dentistry is a relatively new field that has received little attention (Pathak, 2015; Pathak et al., 2015). It is crucial that the aviation sector promotes treatment protocols and diagnostic tools to safeguard the health of its crew, as clinical aeronautic dentistry aims to restore crew members to full wellness after oral emergencies (Pathak, 2015; Pathak et al., 2015), in addition, dental professionals and flight crews should take advantage of any opportunity to incorporate dental and oral health into crews’ physiological prerequisites in order to boost their overall health (Pathak, 2015; Pathak et al., 2015).

Special precautions are necessary for aircrew patients during restorative, endodontic, oral and maxillofacial surgical, and prosthodontics treatments to prevent onboard incapabilities and potential major problems (Pathak, 2015; Pathak et al., 2015; Lloro et al., 2019). Excavating dental decays and restoring them must be performed ahead of flight departure, also leaking fillings ought to get replaced after which an extensive inspection of the cavity floor is necessary to avoid pulp chamber penetration, and a protective cavity liner like glass-ionomer cement is applied. For successful multi-visit root canal therapy, the interim restoration should be appropriately positioned (Pathak, 2015; Pathak et al., 2015). Although, in 1995, root canal therapy for a suspicious pulp chamber invasion in a crew member was needed in order to avoid silent pulp necrosis and sub-acute pulpitis, and their pressure related complications; moreover, when not properly addressed, endodontic infections might result in leaking of diseased tissue into the peri-radicular tissues and subcutaneous emphysema (Niazi and Bakhsh, 2022).

### Preventative measures against dental problems before and during space flights

Cuspal covering indirect restorations can serve as preventative dentistry. Moreover, resin cements are recommended for cementation of indirect restoration due to their superior retention and decreased porosity (Lyons et al., 1997; Pathak, 2015; Pathak et al., 2015). Eating sweets or chewing gum during missions can increases salivary output hence prevent dry mouth; noting that dry mouth is correlated with periodontal diseases and high caries incidence. Once a maxillary tooth is extracted, oral surgeons should check for oro-antral communication, which may cause sinusitis upon exposure to a fluctuating pressure; if present, an oro-antral communication ought to be sutured. Furthermore, osseointegrated dental implants may enhance edentulous denture retention (Pathak, 2015; Pathak et al., 2015).

NASA does not verify an individual onboard prevention technique without considering different bone-loss strategies for each crew. Moreover, pharmaceutical therapies may prevent bone loss during space flights, but prospective studies should be meticulously designed to yield unambiguous results (Orwoll et al., 2013). Finally, in addition to the medical and dental considerations discussed above, it is essential to understand that a holistic approach needs to be taken into account while planning for the health, safety and wellbeing of the astronauts in long-haul space flights. This should take into account the psychological aspects of the astronauts’ health through promotion of positive emotions, subjective resilience and the prevention of the development of emotional disorders to allow adaptation to extreme environments (Gatti et al., 2022). Finally, Table 3 summarises the recommended directions for future research that are deemed necessary to advance the field of aerospace dentistry and provide a holistic approach to the health and wellbeing of astronauts during long-haul space flights.

**TABLE 3:** A summary of the recommended directions for future research in aerospace dentistry.

Field of recommended future research	Aims and objectives
The effect of space flights on teeth/dental hard tissues in humans	• Standardization of factors such as the duration of the space flight, diet, age and sex of the samples • Fill the gaps in literature as most studies in this field are based on rodents’ teeth which are not comparable to human teeth
The importance of prebiotics and probiotics in astronauts’ diet	• Modification and regulation of the microbiome. Helping in lowering levels of inflammatory processes and loss of bone
Effects of microgravity on the maxillary and mandibular alveolar bone	• Confirmation of the extent of the well- established negative effects of microgravity on skeletal bones and correlating them with the alveolar process
Tooth-derived stem cell and oral microbiology research in space	• Further advancement of the fields of regenerative dentistry, medicine, surgical repair and wound healing
Potential negative effects of pharmaceuticals under microgravity environments	• Evaluation of prospective pharmaceutical agents taken for or during space flights • long-term clinical trials for identifying potential low-level negative reactions with denosumab

## Conclusion

This review summarizes the available research with regards to the effects of microgravity and long-haul space flights on oral and dental tissues. We provide a few hypotheses that could contribute to expanding the current available knowledge in this field. Dentistry in space travel is best incorporated within a medical team that provides a holistic and multidisciplinary healthcare approach to the astronauts. Furthermore, the most significant evidence-based changes within the oral cavity that are related to the space environment are changes in the salivary components and oral microbiology. Finally, we highlight the promising potentials for space travel in regenerative medicine and dentistry, especially with regards to the utilization of stem cell culture and bioengineering/biomanufacturing. The information presented in the current review helps provide directions for future research as well as recommendations for maintaining the health of astronauts and preventing the long-term negative effects of space travel and microgravity.
